# Sustainable production of azadirachtin from differentiated *in vitro* cell lines of neem (*Azadirachta indica*)

**DOI:** 10.1093/aobpla/plt034

**Published:** 2013-08-02

**Authors:** Mithilesh Singh, Rakhi Chaturvedi

**Affiliations:** Department of Biotechnology, Indian Institute of Technology – Guwahati, Guwahati 781 039, Assam, India

**Keywords:** *Azadirachta indica*, azadirachtin, HPLC analysis, *in vitro* culture, redifferentiated callus.

## Abstract

Azadirachtin has high industrial demand due to its immediate application as an ecofriendly, biodegradable biopesticide and also due to its various other significant bioactivities. To date, the only commercially feasible way to produce azadirachtin is extraction from seeds, but their availability is very limited as the tree flowers only once a year and only one-third of the fruits are collected due to operational problems. Further, due to the strict out-breeding nature of the plant, the seeds are highly heterozygous, resulting in inconsistent metabolite production. Therefore, in the present study, to achieve sustainable production of azadirachtin, dedifferentiated and redifferentiated calli derived from various explants of neem—zygotic embryo, leaf and ovary—were investigated for their potential to biosynthesize azadirachtin. High-performance liquid chromatography analysis of the *in vitro* cell lines showed the presence of azadirachtin in all the samples tested, the content of which in cultured cells varied with explant source and cell differentiation response. The presence of azadirachtin in samples was further confirmed by positive electrospray ionization mass spectroscopy. The zygotic embryo cultures of neem accumulated much higher amounts of azadirachtin than leaf and ovary cultures. Furthermore, organized *in vitro* callus cultures (redifferentiated) supported higher azadirachtin biosynthesis, while unorganized callus cultures (dedifferentiated) supported the least. The maximum azadirachtin content of 2.33 mg g^−1^ dry weight was obtained from redifferentiated immature zygotic embryo cultures.

## Abstract (precis)

Neem possesses immense medicinal properties and has immediate application as ecofriendly, biodegradable biopesticide. All these properties are because of a structurally complex bioactive compound Azadirachtin, mainly present in seeds. However, it could not be exploited to its full because of heterogeneity in compound production due to out-breeding in nature of the plant. This is the first elaborate report on systematic studies on azadirachtin biosynthesis where different redifferentiated and dedifferentiated *in vitro* cell lines of *A. indica* (neem), obtained from various explants, were utilized. All cell lines were found positive for the compound with significantly higher azadirachtin yield of 2330 µg /g DW was obtained from redifferentiated cell lines established from zygotic embryo cultures. The study, demonstrates the possibility of consistent biosynthesis of azadirachtin in bulk under optimal growth conditions.

## Introduction

Production of secondary metabolites from plant tissue culture has emerged as a promising and feasible option attracting the attention of scientists worldwide. The entire exercise becomes obligatory if we aim at metabolites from an out-breeding tree species, like neem, due to the variability inflicted upon by heterozygosity in the genus. The neem tree has been used from time immemorial in herbal remedies all over the world to cure various ailments. However, in the last two decades, it has become the focus of attention due to its agrochemical, medicinal and economic uses. The tree has been claimed to possess several biological activities, such as immune stimulation, blood purification, anti-inflammation, anti-tumour activity, insect repulsion, bactericidal activity and growth-disrupting properties (Biswas *et al.* 2002; Haque *et al.* 2006). These properties are attributed to several secondary metabolites present in the genus, most of which chemically belong to the class of terpenoids like azadirachtin, nimbin, salannin, margosane and meliacin. Among all, azadirachtin is one of the most complex and important compounds, which has been the focus of research since its isolation and characterization by Butterworth and Morgan (1968).

Azadirachtin is present in all parts of the tree, but its highest concentration lies in mature seeds. All commercial formulations and products based on azadirachtin were prepared by extraction of seeds collected from naturally grown plants. However, this approach has several disadvantages such as heterogeneity in azadirachtin content resulting from seasonal variation and enormous heterozygosity prevalent in the genus due to cross-pollination, its long reproductive cycle, recalcitrant nature and poor seed yields (Ermel *et al.* 1983, 1987; Benge 1989; Schmutterer 1990; Ermel 1995; Sidhu and Behl 1996; Wewetzer 1998; Sidhu *et al.* 2003). Also the geographical distribution of the neem tree is limited. Moreover, neem trees flower once a year, and only about one-third of the seeds are collected due to operational problems and quality considerations. On maturity, the pericarp of a fully ripe fruit contains several carbohydrates in the form of sweet pulp and flesh, which ferment during improper handling and storage (Jayaraj 1993; Vyas and Mistry 1996; Venkateswarlu and Mukhopadhyay 1999). Furthermore, due to the low shelf life of seeds, the azadirachtin percentage dropped to 32 % within 4 months of storage (Yakkundi *et al.* 1995).

In this respect, plant cell and organ cultures offer an attractive alternative for homogeneous, controlled production of metabolites, throughout the year, especially when we take commercial demand into account. They not only facilitate the *de novo* synthesis of novel compounds, but also are able to produce metabolites, sometimes even in higher amounts than the intact plants. Total chemical synthesis could be another route to obtain the required amount of these compounds. However, the structural complexity of azadirachtin, a tetranortriterpenoid, precludes efficient chemical synthesis. Although the synthetic route is established, total chemical synthesis of it is not economically viable (Prakash *et al.* 2002).

The major impediments in neem, with regard to availability of metabolites like azadirachtin, from tissue culture lie in its variable and low productivity. In this study, we made an effort towards systematic selection of explants and screening of *in vitro* cultures for sustainable and improved production of azadirachtin. Redifferentiated and dedifferentiated cultures, established from various explants of neem, were analysed to find the elite cell lines for azadirachtin biosynthesis. No such detailed studies were performed earlier on this aspect. We believe that the present study will give the scientific world a fresh perspective.

## Methods

### Plant material and experimental treatments

All explants were collected from a mature 35-year-old neem tree, growing near the campus of the Indian Institute of Technology Guwahati, Assam, India, during the months of April–June. The basal medium used in all experiments consisted of Murashige and Skoog (MS; 1962) macro- and microsalts, MS vitamins and 100 mg L^−1^ myo-inositol. The pH of the medium was adjusted to 5.8 before autoclaving at 1.06 kg cm^−2^ and 121 °C for 15 min. Cultures were maintained in 1000–2000 lux light intensity at 25 °C and cultured plant cells were harvested between 5 and 6 weeks and dried in an oven at 33 ± 2 °C until a constant weight was achieved. The drying temperature was kept low to prevent thermal decomposition of metabolites.

To establish cultures from zygotic embryo explants, immature fruits were collected and thoroughly washed in 1 % (v/v) savlon (Johnson and Johnson, Solan, Himachal Pradesh, India) solution for 10 min, followed by rinsing with sterile distilled water (SDW). Fruits were then rinsed with 90 % ethanol for 30 s before surface sterilizing, using 0.1 % (w/v) mercuric chloride (HgCl_2_) for 10 min inside a laminar air-flow cabinet (Saveer Biotech, Delhi, India). After washing with SDW three times, the fruits were dissected with the aid of a stereo-microscope (Nikon SMZ-1, Tokyo, Japan). Embryos at early–late dicotyledonary stages were cultured on MS medium supplemented with various combinations of 2,4-dichlorophenoxy acetic acid (2,4-D), *N*^6^-benzylamino purine (BAP) and thidiazuron (TDZ) either alone or in combination with α-naphthalene acetic acid (NAA), indole-3-acetic acid (IAA), *N*^6^-furfuryladenine (kinetin), abscisic acid (ABA), gibberellic acid (GA_3_) and casein hydrolysate (CH), for callus induction and morphogenesis. Four zygotic embryos were placed in one Petri dish with 16 dishes for each treatment. Data were collected as the number of responding zygotic embryos relative to the total number of zygotic embryos cultured. After 5 weeks of culture, the calli were transferred to fresh medium of parental composition for further multiplication and to obtain regeneration of plants from them.

To establish leaf-disc culture, leaves were washed with a mixture of 1 % savlon solution and three drops of Tween-20 (Merck, Mumbai, Maharashtra, India) for 20 min, followed by three rinses in SDW. Thereafter, the remaining steps were carried out inside the laminar air-flow cabinet. Leaves were surface-sterilized with 0.1 % (w/v) HgCl_2_ for 8 min, followed by three rinses in SDW. Leaf-disc explants were prepared by punching the sterilized leaves with a 5-mm-sized cork borer before being cultured with the abaxial side in contact with the medium. Murashige and Skoog basal medium supplemented with different combinations and concentrations of auxins and cytokinins, including BAP, kinetin, zeatin, TDZ, NAA, IAA, indole 3-butyric acid (IBA) and CH, was used to induce callusing from explants. Each experiment contained at least 24 replicates. Data were collected as the number of responding explants relative to the total number of leaf discs cultured. After 5 weeks, leaf-disc calli were transferred to fresh medium of the same composition for further multiplication and shoot regeneration.

For establishing ovary cultures, the flower buds of 4 mm size were sterilized with 0.1 % (w/v) HgCl_2_ for 7 min, followed by three washes in SDW inside the laminar air-flow cabinet. After dissecting the flowers, ovaries were gently taken out and cut transversely into two halves of 0.4–0.5 mm size and implanted with the basal cut end touching the medium. Four ovary slices from two ovaries were placed on each Petri dish with 16 dishes for each treatment. For callus induction, MS medium was tested with 2,4-D or BAP either alone or in combination with kinetin, glutamine and serine. After a pre-treatment at 4 °C and 33 °C for 15 days, the cultures were transferred to 25 °C, to study their callusing response. Some cultures were maintained continuously at 25 °C as a control. Data were collected as the number of responding explants relative to the total number of sliced ovaries cultured. After 4 weeks of culture, ovary calli were transferred to different sets of media combinations for further multiplication and regeneration of shoots from them.

For histological analysis, regenerating calli were sampled and fixed in FAA (5 : 5 : 90, v/v/v formalin : acetic acid : 70 % ethanol) for 48 h and stored in 70 % alcohol. The material was passed through a tertiary-butyl alcohol series for dehydration, infiltrated with paraffin wax (melting point 60 °C; E. Merck, Darmstadt, Hesse, Germany) and embedded in pure paraffin wax, and 8- to 10-µm-thick sections were cut using a rotary microtome (Leica, Solms, Hesse, Germany). The sections were mounted on microslides, dewaxed and double-stained with safranin (1 %) and astra-blue (1 %) to trace the developing vascular strands in the tissues.

### Analysis of *in vitro* cell lines for azadirachtin production

Azadirachtin standard was procured from Sigma Aldrich (St Louis, MO, USA) and dissolved in high-performance liquid chromatography (HPLC) grade methanol (Merck) to yield a stock concentration of 1000 µg mL^−1^. The stock solution was serially diluted with HPLC grade methanol to make samples with concentrations of 250, 125, 62.5, 31.3, 15.6 and 7.8 µg mL^−1^. Each concentration of standard was filtered through 0.22-µm nylon membrane filters (Millipore, Billerica, MA, USA) before HPLC analysis.

To prepare samples, cultured plant cells were harvested from various media, washed with distilled water and filtered under vacuum. Thereafter, washed cell lines and seed, leaf and ovary samples were dried separately in an oven at 33 ± 2 °C until a constant weight was achieved. The drying temperature was kept low to prevent thermal decomposition of metabolites. The dried samples were dipped in methanol overnight and, thereafter, sonicated for 45 min at 35 % amplitude with 5-s pulses on and off. Samples were centrifuged in a high-speed refrigerated centrifuge (Sigma 4K15C, Osterode Am Harz, Göttingen, Lower Saxony, Germany) at 5000 rpm for 10 min. The supernatant was pooled and water was added in the ratio of 40 : 60 (40 % water and 60 % methanol). After addition of water, the solution was partitioned against 100 mL of dichloromethane (DCM) in separating funnels. The solution was mixed thoroughly and the separating funnels were kept aside for 10 min to separate two immiscible solvents (methanol + water and DCM). Later, the upper water–methanol layer was discarded and the DCM layer was collected and then evaporated to dryness at 40 °C temperature in a rotary vacuum evaporator (Buchi Rotavapor R-200, Tokyo, Japan). The DCM fraction residue thus obtained was redissolved in HPLC grade methanol and filtered through a 0.22-µm nylon membrane filter prior to analysis and aliquots of 20 µL of clean solution were injected into the HPLC system. Extraction from seeds consisted of an additional step of defatting with hexane prior to extraction with methanol.

High-performance liquid chromatography was conducted for quantitative estimation of azadirachtin using the Varian Prostar HPLC system (Varian, Palo Alto, CA, USA) equipped with a UV–visible spectrophotometer detector, a prostar binary pump, a 20-µL injection loop and a Hypersil BDS RP-C18 column (Thermo, Waltham, MA, USA) of dimensions 250 × 4.6 mm. The mobile phase used was 90 % methanol and 10 % water at a flow rate of 0.5 mL min^−1^. Ultraviolet detection was carried out at 210 nm with an attenuation of 0.1 absorbance units at full scale. The chromatographic peaks of the analytes were confirmed by comparing their retention times with those of the azadirachtin standard (≈95 %).

The linearity of the developed method was checked by running the standard compound at different concentrations. A calibration curve was generated by plotting the peak area (*y*) against concentration in µg mL^−1^ of standard solutions (*x*) on Microsoft Office (Excel) Professional Edition 2003 (Microsoft, Redmond, WA, USA). The standard equation obtained from the curve was used for quantification of the compound in unknown samples. The correlation coefficients (*R*^2^) were also generated in Excel by fitting the linear trendlines to the standard curve obtained. Precision of the developed assay was evaluated by running the same concentration of standard compounds at least three times on the same day (intraday) and twice at 1-day intervals (interday). The values were calculated in terms of per cent relative standard deviation (% RSD), which is calculated as (standard deviation/mean) × 100.

Mass spectroscopy detection was carried out on a Waters quadrapole-Tof premier mass spectrometer with a microchannel plate detector (Waters, Milford, MA, USA). Samples were analysed in positive mode with a probe temperature of 400 °C and a source block temperature of 150 °C. The source was operated with a corona pin voltage of 3.50 kV and a cone voltage of 25 V. The MS data were obtained in full scan mode (mass range 100–1000 amu). A comparison of mass spectra of the standard compound obtained from Sigma, with that of the sample isolated from HPLC, confirmed the presence of azadirachtin.

### Statistical analysis

Data were subjected to analysis of variance (ANOVA) and means were compared using Duncan's multiple range test by SPSS 16.0 software. *P* values <0.05 were considered statistically significant. Arcsine transformation was done for the per cent values before proceeding for ANOVA. For azadirachtin estimation, all results are an average of three separate analyses, each having five replicates. Results are represented as mean ± standard deviation.

## Results

### Establishment of dedifferentiated and redifferentiated cultures

**Figure 1.**
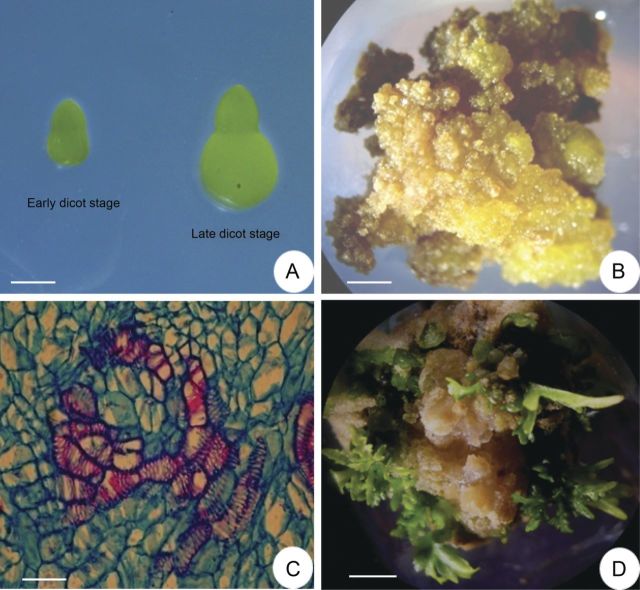
Callus induction and shoot regeneration from zygotic embryo cultures. (A) Early and late dicotyledonary stages of embryos at the time of culture (scale bar = 0.05 cm). (B) A 4-week-old dedifferentiated callus proliferated from zygotic embryo explants on MS + BAP (5.0 µM) + 2,4-D (1.0 µM) + NAA (1.0 µM) medium (scale bar = 0.4 cm). (C) Histological section of a nodulated 5-week-old callus culture on MS + BAP (9.0 µM) + IAA (5.0 µM) + CH (500 mg L^−1^), showing vascular strands within the calli (scale bar = 25 µm). (D) A 5-week-old callus subculture of (C), showing shoot proliferation and well-developed shoots (scale bar = 0.59 cm).

To establish cultures, zygotic embryos at early–late dicotyledonary stages (Fig. 1A) were cultured on MS basal medium variously supplemented with growth regulators. Murashige and Skoog basal medium alone did not show any response. Of the various treatments tested, dedifferentiated (non-regenerative) cultures were obtained from the late dicotyledonary stage of embryos on MS + BAP (5.0 mM) + 2,4-D (1.0 mM) + NAA (1.0 mM) (Table 1). On this medium, within 4 weeks, profusely growing light green, fresh and friable calli proliferated from 100 % explants (Fig. 1B). The callus proliferation rate was increased in subsequent subcultures on the same medium. However, the calli remained in the dedifferentiated state. For redifferentiated cultures, MS + BAP (9.0 mM) + IAA (5.0 mM) + CH (500 mg L^−1^) medium was found to be the best (Table 1). On this medium, calli proliferated within 1 week in 78 % of cultures, which after 5 weeks turned hard and nodulated. Histological analysis of nodulated callus cultures showed the development of vascular strands within the calli (Fig. 1C). On subsequent subcultures of calli on the same medium, shoot proliferation started from these calli, within 3 weeks (Fig. 1D). Both the cell lines, dedifferentiated and redifferentiated, were maintained for >3 years.

**Table 1.** Effect of various growth regulators, alone or in combination, on callusing response from zygotic embryo explants of neem. Growth period: 5 weeks. Control: MS basal medium. Mean values sharing the same letter do not differ significantly (*P* < 0.05) according to Duncan's multiple range test.

Sl. no.	Media	Per cent callusing response
1	MS basal medium	0.0l
2	MS + BAP (5.0 µM)	35.2g
3	MS + TDZ (5.0 µM)	13.4j
4	MS + 2,4-D (5.0 µM)	0.0l
5	MS + BAP (5.0 µM) + ABA (1.0 µM)	18.9i
6	MS + TDZ (5.0 µM) + ABA (1.0 µM)	35.8g
7	MS + 2,4-D (5.0 µM) + ABA (1.0 µM)	0.0l
8	MS + BAP (5.0 µM) + GA_3_ (1.0 µM)	0.0l
9	MS + TDZ (5.0 µM) + GA_3_ (1.0 µM)	10.5k
10	MS + 2,4-D (5.0 µM) + GA_3_ (1.0 µM)	0.0l
11	MS + BAP (5.0 µM) + 2,4-D (1.0 µM)	55.6e
12	MS + BAP (5.0 µM) + 2,4-D (1.0 µM) + NAA (1.0 µM)	100a
13	MS + BAP (5.0 µM) + 2,4-D (1.0 µM) + CH (500 mg L^−1^)	54.3e
14	MS + BAP (5.0 µM) + 2,4-D (5.0 µM) + CH (500 mg L^−1^)	42.9f
15	MS + BAP (9.0 µM) + 2,4-D (1.0 µM)	30.3h
16	MS + BAP (9.0 µM) + 2,4-D (1.0 µM) + NAA (1.0 µM)	67.3c
17	MS + BAP (9.0 µM) + 2,4-D (1.0 µM) + CH (500 mg L^−1^)	27.9h
18	MS + BAP (9.0 µM) + 2,4-D (5.0 µM) + CH (500 mg L^−1^)	20.3i
19	MS + BAP (5.0 µM) + IAA (1.0 µM)	19.7i
20	MS + BAP (5.0 µM) + IAA (1.0 µM) + CH (500 mg L^−1^)	12.3j
21	MS + BAP (5.0 µM) + IAA (5.0 µM) + CH (500 mg L^−1^)	27.5h
22	MS + BAP (9.0 µM) + IAA (1.0 µM)	43.7f
23	MS + BAP (9.0 µM) + IAA (1.0 µM) + CH (500 mg L^−1^)	60.3d
24	MS + BAP (9.0 µM) + IAA (5.0 µM) + CH (500 mg L^−1^)	78.0b

Leaf-disc explants of 5 mm size were cultured on a range of media that involved MS basal medium and varying concentrations and combinations of cytokinins and auxins (Table 2). Leaf-disc cultures showed no response in the absence of growth regulators. The presence of at least one cytokinin or one auxin was found to be obligatory for caulogenic induction.

**Table 2.** Effect of various growth regulators, alone or in combination, on callusing response from leaf-disc explants of neem. Growth period: 5 weeks. Control: MS basal medium. Mean values sharing the same letter do not differ significantly (*P* < 0.05) according to Duncan's multiple range test.

Sl. no.	Media	Per cent callusing response
1	MS basal medium	0.0i
2	MS + BAP (5.0 µM)	76.0c
3	MS + TDZ (5.0 µM)	40.0e
4	MS + kinetin (5.0 µM)	0.0i
5	MS + zeatin (5.0 µM)	100a
6	MS + 2,4-D (5.0 µM)	19.7g
7	MS + NAA (5.0 µM)	84.7b
8	MS + IAA (5.0 µM)	0.0i
9	MS + IBA (5.0 µM)	100a
10	MS + BAP (2.2 µM) + NAA (21.5 µM) + CH (500 mg L^−1^)	100a
11	MS + BAP (5.0 µM) + 2,4-D (1.0 µM) + NAA (1.0 µM)	11.3h
12	MS + BAP (2.0 µM) + 2,4-D (5.0 µM)	39.7e
13	MS + 2,4-D (5.0 mM) + CH (1000 mg L^−1^)	29.7f
14	MS + BAP (2.0 µM) + NAA (5.0 µM)	20.3g
15	MS + BAP (2.0 µM) + NAA (5.0 µM) + CH (500 mg L^−1^)	47.9d

Among individual cytokinin/auxin treatments, MS + zeatin (5.0 µM) and MS + IBA (5.0 µM) evoked maximum callus induction (100 %) and produced brownish-green calli from all over the surface of the explants within 5 weeks. Individually, TDZ and NAA at 5.0 µM concentration also responded to callus induction and produced slow-growing yellowish-brown calli. However, none of these treatments exhibited any kind of organogenesis. Moreover, in subcultures on the original media, the calli did not show any sustained growth and the rate of callus proliferation declined after three subcultures, each of 5 weeks duration. Leaf-disc explants showed no response on kinetin- or IAA-supplemented medium.

**Figure 2.**
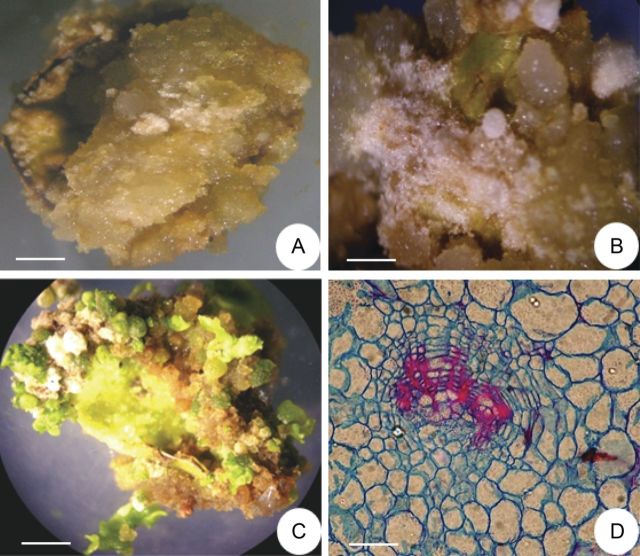
Callus induction and shoot regeneration from leaf-disc cultures. (A) A 3-week-old culture on MS + BAP (2.2 µM) + NAA (21.5 µM) + CH (500 mg L^−1^), showing light brown, fresh and friable calli proliferated from entire leaf-disc explants (scale bar = 0.11 cm). (B) Same as (A), after 5 weeks, showing profuse callus growth (scale bar = 0.17 cm). (C) A 3-week-old culture of leaf discs on MS + BAP (5.0 µM), showing shoot differentiation from brown and dark green, nodulated, compact calli developed at the cut end of the explant (scale bar = 0.11 cm). (D) Histological analysis of a regenerating culture from (C), showing compactly arranged cells and tracheids (33 µm).

The interactive effect of auxin and cytokinin has also been evaluated. The combined presence of one auxin and one cytokinin supported sustained callus growth. The best treatment for callusing, in terms of the number of explants callused (100 %) and the degree of callusing, was MS + BAP (2.2 µM) + NAA (21.5 µM) + CH (500 mg L^−1^). On this medium, callus growth was significant (*P* < 0.05). The fast-growing, fresh and friable calli were obtained in 5 weeks. These calli remained dedifferentiated even after 17 subcultures, each of 5 weeks duration (Fig. 2A and B).

Organogenesis from leaf-disc explants was achieved on MS medium supplemented with BAP alone at 5.0 µM. On this medium, brownish-green and compact calli were developed at the cut end of the explants, within 3 weeks, which later turned into brown and dark green, nodulated calli after 5 weeks of culture initiation, in 76 % of the cultures. On subculture to the original medium, the nodular structures organized into well-developed shoots within 3 weeks (Fig. 2C); an average of seven green shoots was developed per explant. Histological sections of the regenerating calli, passing through the nodules, showed the presence of developing vascular strands within the tissue, indicating the points of origin of shoot buds. A nest of vascular bundles can be seen in the section (Fig. 2D).

**Figure 3.**
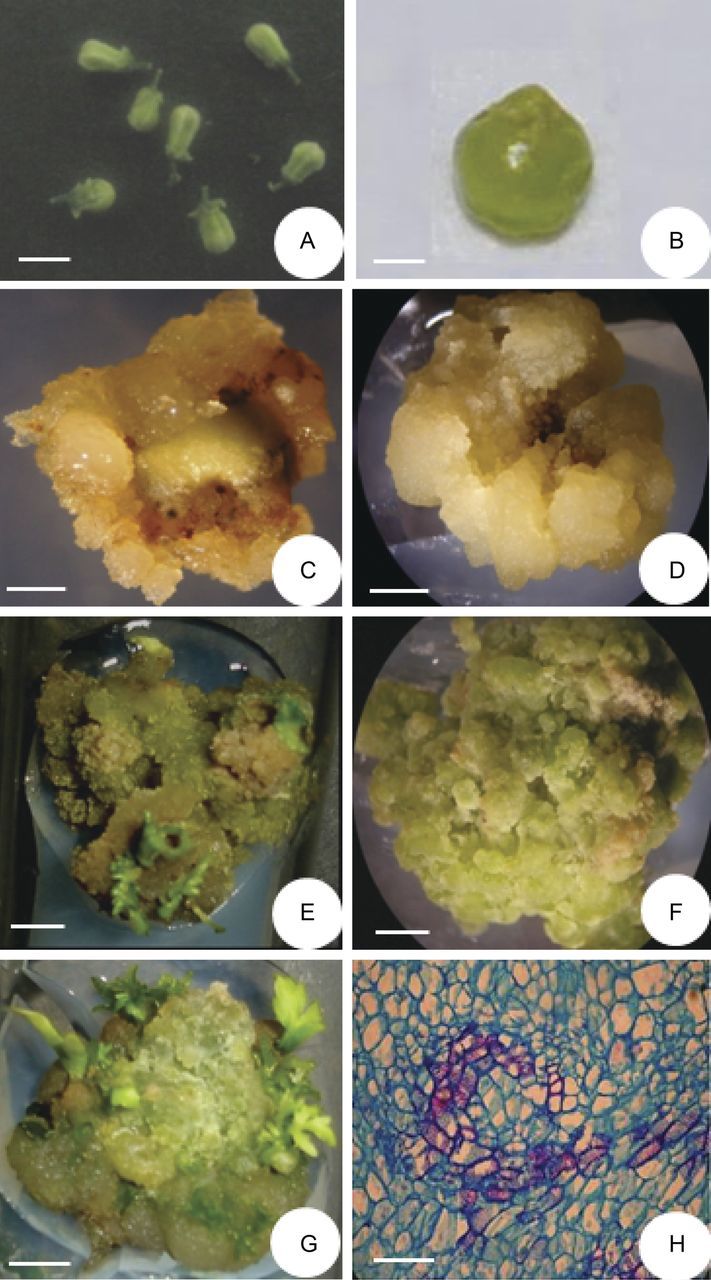
Callus induction and shoot regeneration ovary culture. (A) Flower buds of 4 mm size used for ovary culture (scale bar = 0.7 cm). (B) An excised ovary from 4 mm flower buds (scale bar = 0.05 cm). (C) A 2-week-old ovary slice culture on MS + 2,4-D (0.5 µM) + kinetin (4.5 µM), showing emergence of cream, fresh and friable calli from cut regions (scale bar = 0.2 cm). (D) Same as (C), after 4 weeks, where the entire explant is covered with the cream, friable and fast-growing callus (scale bar = 0.37 cm). (E) A 4-week-old callus subculture on MS + BAP (5.0 µM) + IAA (0.5 µM), showing shoot proliferation (scale bar = 0.35 cm). (F) A 4-week-old bright green, compact callus on MS + BAP (9.0 µM) + IAA (5.0 µM) + CH (500 mg L^−1^) (scale bar = 0.4 cm). (G) Same as (F), 4 weeks after subculture to the same medium, showing differentiation of shoots from dark green, compact nodular regions (scale bar = 0.52 cm). (H) Histological section of a regenerating ovary callus, showing well-developed tracheids (scale bar = 30 µm).

As an alternative approach for secondary metabolite production, the *in vitro* ovary culture method was followed. For initiation of ovary cultures, flower buds of 4 mm size (Fig. 3A) were selected, ovaries were excised (Fig. 3B) and either 0.4- to 0.5-mm-thick transverse sections of ovaries or entire ovaries were cultured on MS basal medium or basal medium supplemented with a range of growth regulators. To induce callusing from explants, cultures were exposed to low- (4 °C) and high-temperature (33 °C) treatments for 0 or 15 days prior to their transfer to standard culture room conditions. Exposure of slice sections at 33 °C was found to be very effective for caulogenic induction. However, slice sections exposed at 4 °C did not show any response (Table 3). In the control (25 °C), initially explants showed small callusing and after the first subculture calli turned brown without showing any further proliferation. In all cases, the response of ovary slice cultures was shown to be better than that of entire ovary cultures, in terms of time taken for callus initiation and degree of callusing. In slice cultures, callusing started after 1 week and explants were completely covered with the proliferating callus by the end of the fourth week (Fig. 3C and D), while for the entire ovary culture it took 4 weeks to start callus initiation.

**Table 3.** Effect of temperature pre-treatments and different growth regulator combinations on per cent callus induction from ovary slice cultures. Growth period: 4 weeks. Control: MS basal medium. Mean values sharing the same letter do not differ significantly (*P* < 0.05) according to Duncan's multiple range test.

Media	Temperature pre-treatments		
	25 °C (control)	4 °C	33 °C
MS basal medium	0.0d	0.0	0.0e
MS + 2,4-D (0.5 µM)	36.7a	0.0	86.5b
MS + 2,4-D (0.5 µM) + kinetin (4.5 µM)	38.2a	0.0	89.0ab
MS + 2,4-D (1.0 µM) + kinetin (10.0 µM)	30.3b	0.0	50.3d
MS + 2,4-D (0.5 µM) + kinetin (4.5 µM) + glutamine (800 mg L^−1^) + serine (200 mg L^−1^)	38.3a	0.0	92.3a
MS + BAP (5.0 µM) + glutamine (800 mg L^−1^) + serine (200 mg L^−1^)	21.4c	0.0	77.3c

Of the various growth regulator combinations tested, MS + 2,4-D (0.5 mM) + kinetin (4.5 mM) + glutamine (800 mg L^−1^) + serine (200 mg L^−1^) induced callusing in the maximum (92.3 %) number of cultures during the initial period of 4 weeks but did not promote sustained proliferation of calli in successive subcultures. In terms of biomass and sustained rate of callus proliferation, the best response was observed on MS + 2,4-D (0.5 µM) + kinetin (4.5 µM) followed by MS + 2,4-D (0.5 µM) and MS + 2,4-D (1.0 µM) + kinetin (10.0 µM), in a single growth cycle of 4 weeks. Moderate- to fast-growing, creamy, soft and friable calli obtained on these three combinations were multiplied and maintained for >1 year.

Since calli on the above three multiplication media remained dedifferentiated, to achieve regeneration, ovary calli from the above three media were transferred to MS medium comprising different combinations and concentrations of BAP, IAA and CH. Out of the three multiplication media, the calli from MS + 2,4-D (0.5 µM) + kinetin (4.5 µM) only showed regeneration in regeneration media and shoot regeneration was achieved on only two combinations: MS + BAP + IAA or MS + BAP + IAA + CH (Table 4). The calli in the regeneration media initially turned bright green in colour and later on within 4 weeks compact nodular structures developed that after subculture to the same medium differentiated into shoot buds in the next 4 weeks. On the first combination, MS + BAP (5.0 µM) + IAA (0.5 µM), on average 25.5 % of cultures formed 2.5 shoot buds per explant in 4 weeks (Fig. 3E). In comparison with this, the second medium, MS + BAP (9.0 µM) + IAA (5.0 µM) + CH (500 mg L^−1^), was found to be the best for shoot regeneration where 50 % of the callus cultures developed on average 5.5 shoot buds per explant in 4 weeks (Fig. 3F and G). Histological analysis of regenerating calli showed the presence of well-formed tracheary elements (Fig. 3H).

**Table 4.** Effect of three induction media on shoot-bud differentiation from ovary calli in the regeneration media. Growth period: 4 weeks. Control: MS basal medium. ^a^No response. ^b^Values in parentheses represent average number of cultures showing shoot regeneration.

Regeneration treatments (µM)	Induction media		
	MS + 2,4-D (0.5 µM)	MS + 2,4-D (0.5 µM) + kinetin (4.5 µM)	MS + 2,4-D (1.0 µM) + kinetin (10.0 µM)
MS basal medium	^a^	^a^	^a^
MS + CH (500 mg L^−1^)	^a^	^a^	^a^
MS + BAP (5.0 µM)	Bright green, friable callus	Bright green, friable callus	Bright green, friable callus
MS + BAP (9.0 µM)	Bright green, friable callus	Bright green, friable callus	Bright green, friable callus
MS + BAP (9.0 µM) + IAA (5.0 µM)	Bright green, friable callus	Bright green, friable callus	Bright green, friable callus
MS + BAP (5.0 µM) + IAA (0.5 µM)	Dark green, friable callus	Bright green, moderately hard; fresh nodulated callus with shoot buds (25.5)^b^	Dark green, friable callus
MS + BAP (9.0 µM) + IAA (5.0 µM) + CH (500 mg L^−1^)	Bright green, moderately hard callus	Bright green, with massive growth, moderately hard; fresh nodulated callus with shoot buds (50)^b^	Bright green, moderately hard callus

### Analysis of *in vitro* cell lines for azadirachtin production

To investigate the effect of explant source and shoot morphogenesis on azadirachtin production, the best three redifferentiated and three dedifferentiated calli obtained from three explants of neem, viz*.* zygotic embryo, leaf and ovary, were analysed by HPLC.

**Figure 4.**
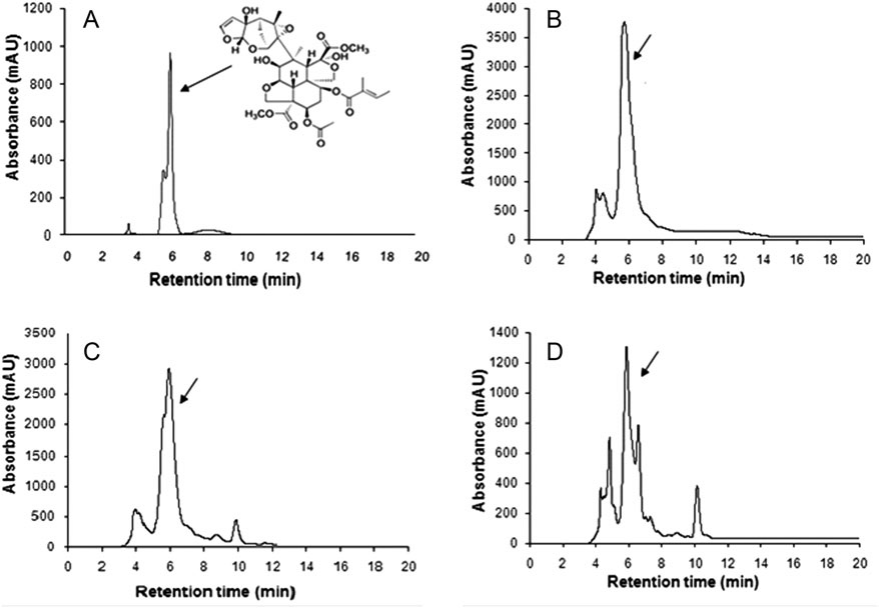
Chromatograms of standard and control samples. Chromatographic profile of (A) standard azadirachtin (marked by an arrow), (B) *in vivo* seed extract showing an azadirachtin peak (control I), (C) *in vivo* leaf extract showing an azadirachtin peak (control II) and (D) *in vivo* ovary extract showing an azadirachtin peak (control III).

With the protocol adopted, an azadirachtin peak was obtained at a retention time of 6.0 ± 0.39 min (Table 5, Fig. 4A). The calibration curve for azadirachtin standard showed good linearity with high reproducibility and accuracy at all the tested concentrations (7.8–250 µg mL^−1^). Regression analysis of the calibration curve data points showed an excellent correlation coefficient (*R*^2^) of 0.9638. The linear regression equation for standard azadirachtin was *y* = 1.0194*x* + 54.22, where *x* is the concentration of standard and *y* is the total peak area. The equation thus generated from the curve by the external standard method was used to calculate the amount of compound present in crude samples.

**Table 5.** Standard curve analysis for azadirachtin.

Retention time (min) ± SD	6.0 ± 0.39
Standard equation	*y* = 1.0194*x* + 54.22
*R*^2^	0.9638
% RSD	
Interday	3.14
Intraday	2.48

The precision of the developed method, as mentioned in Methods, was evaluated by measuring intra- and interday variability in terms of RSD. The standard samples, at the same concentration, were analysed at least three times within the same day and the RSD value obtained was 2.48 %. Similarly, for interday variability, the same concentration of the standard was run at least twice at 1-day intervals and the RSD values were found to be 3.14 % (Table 5).

**Figure 5.**
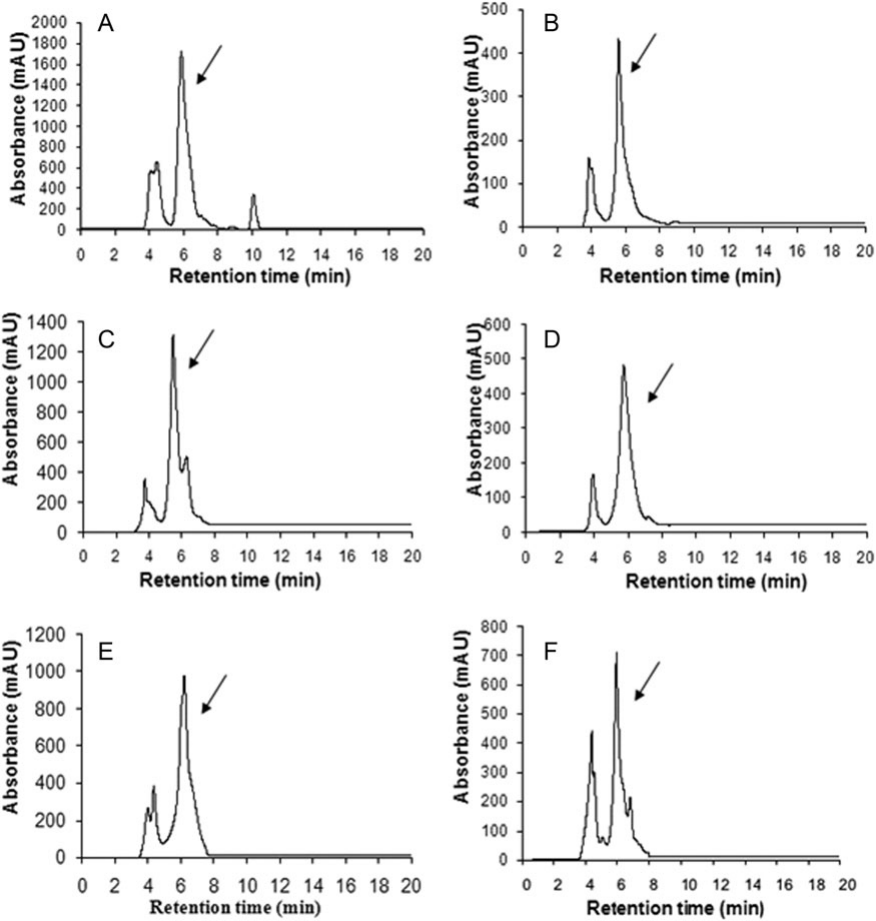
Chromatograms of callus lines. (A) Chromatogram of *in vitro* redifferentiated zygotic embryo calli showing an azadirachtin peak (arrow). (B) Chromatogram of *in vitro* dedifferentiated zygotic embryo calli showing an azadirachtin peak (arrow). (C) Chromatogram of *in vitro* redifferentiated leaf calli showing an azadirachtin peak (arrow). (D) Chromatogram of *in vitro* dedifferentiated leaf calli showing an azadirachtin peak (arrow). (E) Chromatogram of *in vitro* redifferentiated ovary calli showing an azadirachtin peak (arrow). (F) Chromatogram of *in vitro* dedifferentiated ovary calli showing an azadirachtin peak (arrow).

Identification and quantification of azadirachtin were done by HPLC. By following the protocol described in Methods, three controls, viz. seeds (control I), leaves (control II) and ovaries (control III), and three dedifferentiated and three redifferentiated cell lines, obtained from explants, were analysed for azadirachtin accumulation. The presence of azadirachtin was confirmed in all the control samples (Fig. 4B–D) and cell lines (Fig. 5A–F) by the HPLC peak obtained at 6.0 ± 0.39 min retention time.

From the standard equation obtained, the amount of azadirachtin in different callus lines was calculated and is listed in Table 6. The estimation of azadirachtin in different control samples revealed that the seed extract contained azadirachtin (7.41 ± 0.07 mg g^−1^ dry weight (DW)) in amounts that were orders of magnitude higher than those in the leaf (5.49 ± 0.08 mg g^−1^ DW) and ovary (1.38 ± 0.02 mg g^−1^ DW) extracts. Among *in vitro* lines, the highest azadirachtin production (2.33 ± 0.03 mg g^−1^ DW) occurred in redifferentiated zygotic embryo calli, while the least (0.52 ± 0.01 µg g^−1^ DW) was obtained from dedifferentiated leaf calli (Table 6).

**Table 6.** Effect of explant source and organogenesis on azadirachtin content. The values are mean ± SD; mean values sharing the same letter do not differ significantly (*P* < 0.05) according to Duncan's multiple range test.

Source	Medium	Culture (5 weeks old)	Amount of azadirachtin (mg g^−1^ DW) ± SD
Zygotic embryo	Seed (control I)		7.41 ± 0.07a
	MS + BAP (9.0 µM) + IAA (5.0 µM) + CH (500 mg L^−1^)	Redifferentiated	2.33 ± 0.03c
	MS + BAP (5.0 µM) + 2,4-D (1.0 µM) + NAA (1.0 µM)	Dedifferentiated	1.15 ± 0.01g
Leaf	Leaf (control II)		5.49 ± 0.0b
	MS + BAP (5.0 µM)	Redifferentiated	1.6 ± 0.01d
	MS + BAP (2.2 µM) + NAA (21.5 µM) + CH (500 mg L^−1^)	Dedifferentiated	0.52 ± 0.01i
Ovary	Ovary (control III)		1.38 ± 0.02e
	MS + BAP (9.0 µM) + IAA (5.0 µM) + CH (500 mg L^−1^)	Redifferentiated	1.28 ± 0.02f
	MS + 2,4-D (0.5 µM) + kinetin (4.5 µM)	Dedifferentiated	1.03 ± 0.01h

**Figure 6.**
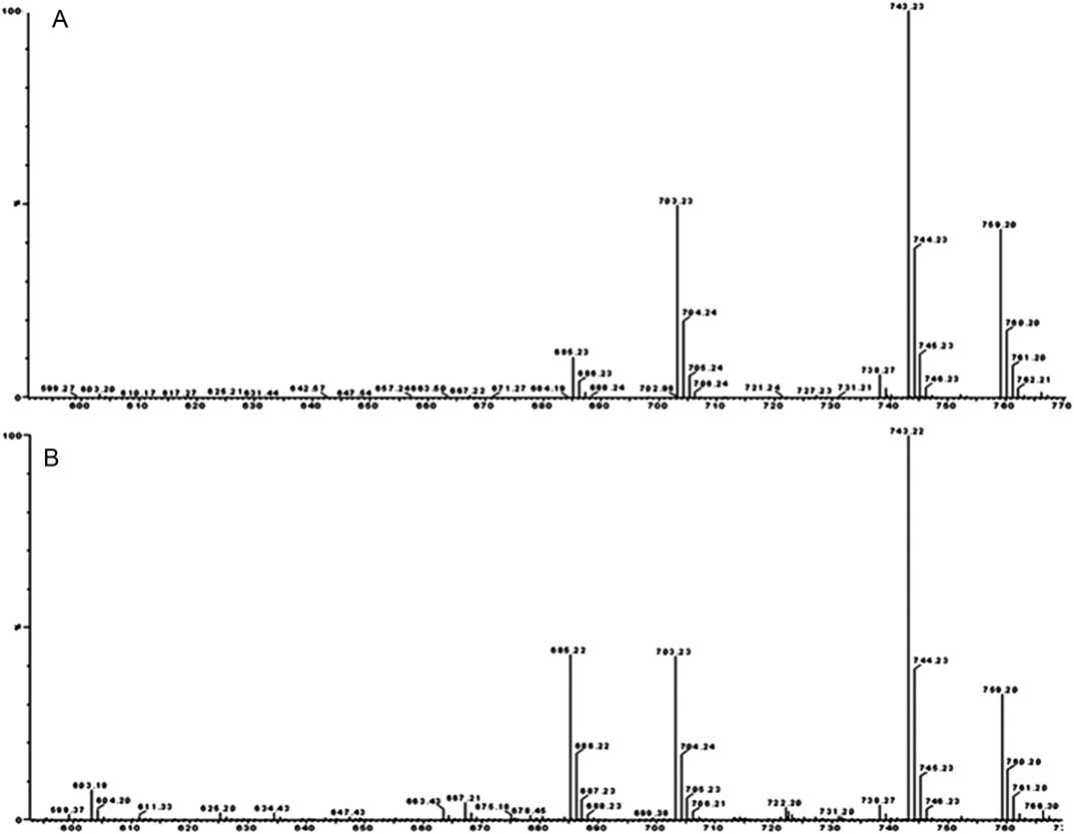
Mass spectrometric profile of (A) standard azadirachtin and (B) HPLC eluted fraction of crude extract obtained from one of the best calli lines on MS + BAP (9.0 µM) + IAA (5.0 µM) + CH (500 mg L^−1^) medium.

The fraction of crude extracts eluted from HPLC at a retention time of 6.0 ± 0.39 min was collected and analysed by mass spectrometry, and the fragment characteristics were compared with those of standard azadirachtin procured from Sigma-Aldrich. Spectra were obtained in full scan mode. The positive electrospray ionization mass spectrum has a base peak at *m/z* 743 corresponding to the [M + Na]^+^ ion. The ions at *m*/*z* 759 were formed due to potassium ion adduct formation [M + K]^+^. Characteristic fragments seen at *m*/*z* 685 and *m*/*z* 703 may be due to elimination of water, [MH − 2H_2_O]^+^ and [MH − H_2_O]^+^, respectively (Fig. 6A and B). Similar *m*/*z* fragments in the standard compound and the HPLC fraction confirmed the presence of azadirachtin in *in vitro* cell lines.

## Discussion

During the past decades, concerted efforts have been made to establish *in vitro* cultures of neem and success has been achieved merely in the field of establishment of large-scale plantations. However, the full potential of neem tissue culture as a means of producing metabolite is limited owing to the fact that in most studies, established cultures were not analysed for metabolite production. Since the plant is highly heterozygous due to cross-pollination, considerable gains in consistent and homogeneous production of azadirachtin can be achieved by utilizing *in vitro* cultures.

In this study, all redifferentiated and dedifferentiated *in vitro* cell lines of neem showed the presence of azadirachtin, the content of which varied with explant source and cell differentiation response. In agreement with the report on localization of azadirachtin in seeds (Govindachari *et al.* 1992), the zygotic embryo cultures of neem accumulated much higher (2.33 mg g^−1^ DW) amounts of azadirachtin than leaf and ovary cultures. Furthermore, the results of this study revealed that organized *in vitro* callus cultures (redifferentiated) supported higher azadirachtin biosynthesis, while unorganized callus cultures (dedifferentiated) supported the least. It has been observed that BAP alone or with IAA and CH promoted shoot organogenesis in all cultured explants that stimulated an increase in azadirachtin production. The effect of growth regulators on secondary metabolite production has also been very well documented (Anuradha *et al.* 2010; Nagella and Murthy 2010; Lee *et al.* 2011).

Under *in vitro* conditions, redifferentiation is generally associated with an improved synthesis of secondary metabolites (Collin 2001). This is most probably due to the appearance of complex cells and tissues that are metabolically more proficient. In all redifferentiated cell lines along with the shoot-forming nodules, non-morphogenic cell masses were also present, which although non-morphogenic might have a certain degree of differentiation at the cellular stage and, due to co-evolution, imitated the biochemistry of redifferentiated cells (Brown and Charlwood 1986). Further, the results of the present study are in agreement with the report on *Artemisia annua*, where artemisinin production was very poor in dedifferentiated callus cultures and a certain degree of redifferentiation was obligatory for artemisinin production (Liu *et al.* 1997). Organogenesis was also found to be an essential prerequisite for steroidal saponin production in *Ruscus aculeatus* (Palazón *et al.* 2006). Similar observations were made for the biosynthesis of picroside in *Picrorhiza kurroa*, wherein the metabolite did not accumulate in the dedifferentiated callus cultures but occurred specifically in the redifferentiated cultures (Sood and Chauhan 2009). Berkov *et al.* (2010) also demonstrated that alkaloid synthesis in *Pancratium maritimum* is closely related to tissue differentiation.

The available reports on azadirachtin production have utilized *in vitro* cultures from different parts of the plant, like leaves (Allan *et al.* 1994, 2002; Kearney *et al.* 1994; Sundaram and Curry 1996; Wewetzer 1998; Kuruvilla *et al.* 1999), bark (Wewetzer 1998), embryos (Srividya *et al.* 1998), seeds (Prakash *et al.* 2005), shoot tips (Schaaf *et al.* 2000), shoots (Raval *et al.* 2003) and microspore-derived calli (Srivastava and Chaturvedi 2011). Whereas in leaf-derived calli, the amount ranged from 4 to 64 µg g^−1^ DW in different reports, bark, embryo, shoot, shoot tip and microspore-derived cultures produced 44–1200, 4–8, 250, 0.5 and 728.41 µg g^−1^ DW of azadirachtin, respectively. In the present study, azadirachtin yield (2330 µg g^−1^ DW) obtained from redifferentiated zygotic embryo cultures is significantly higher than the highest azadirachtin yielding dedifferentiated seed-derived cell line (1890 µg g^−1^ DW) established by Prakash *et al.* (2005).

Azadirachtin production in this study is much higher than the literature-reported values of other investigations except for that of Prakash and Srivastava (2005, 2008), wherein higher azadirachtin production was reported after optimization of media constituents and elicitors. It is believed that the 2330 µg g^−1^ DW (2.33 mg g^−1^ DW) of azadirachtin value, obtained in the present study, will be improved significantly after optimization and elicitation. This study, thus, demonstrates the possibility of biosynthesis of azadirachtin in bulk under proper growth conditions. The literature suggests that reports on systemic selection and screening of *in vitro* cell lines for azadirachtin production are lacking. In this context, the present study will be of significant importance that can be applied to scale up and maximize azadirachtin production in cell, tissue and organ cultures of neem.

## Conclusions

Taken as a whole, our results suggest that shoot organogenesis is important for the biosynthesis of azadirachtin and that dedifferentiated tissues have a limited capacity to produce this compound. Among the analysed cell lines, the highest amount of azadirachtin (2.33 mg g^−1^ DW) was accumulated in redifferentiated zygotic embryo cultures of neem. This elite cell line can be used for sustainable production of azadirachtin. In future, this system may also be helpful in understanding the biosynthetic mechanism of azadirachtin synthesis under *in* *vitro* conditions.

## Sources of Funding

Our work was funded by the Department of Science and Technology (DST), New Delhi, India.

## Contributions by the Authors

The work presented here was carried out in collaboration between both the authors.

## Conflict of Interest Statement

None declared.
